# Differential arthritogenicity of *Staphylococcus aureus* strains isolated from biological samples

**DOI:** 10.1186/1471-2334-13-400

**Published:** 2013-08-30

**Authors:** Priscila Maria Colavite-Machado, Larissa Lumi Watanabe Ishikawa, Thaís Graziela Donegá França, Sofia Fernanda Gonçalves Zorzella-Pezavento, Larissa Camargo da Rosa, Fernanda Chiuso-Minicucci, Maria de Lourdes Ribeiro de Souza da Cunha, Gustavo Pompermaier Garlet, Alexandrina Sartori

**Affiliations:** 1 Department of Microbiology and Immunology, Biosciences Institute, Univ. Estadual Paulista (UNESP), Distrito de Rubião Júnior s/n, 18618-070 Botucatu, São Paulo, Brazil; 2 Department of Biological Sciences, School of Dentistry of Bauru, São Paulo University-FOB/USP, Bauru, São Paulo, Brazil

**Keywords:** Staphylococcus aureus, Septic arthritis, IL-17

## Abstract

**Background:**

*Staphylococcus aureus* is the most common agent of septic arthritis that is a severe, rapidly progressive and destructive joint disease. Superantigens produced by *S. aureus* are considered the major arthritogenic factors. In this study, we compared the arthritogenic potential of five superantigen-producing staphylococcal strains.

**Methods:**

Male C57BL/6 mice were intravenously infected with ATCC 19095 SEC^+^, N315 ST5 TSST-1^+^, S-70 TSST-1^+^, ATCC 51650 TSST-1^+^ and ATCC 13565 SEA^+^ strains. Clinical parameters as body weight, arthritis incidence and clinical score were daily evaluated. Joint histopathological analysis and spleen cytokine production were evaluated at the 14th day after infection.

**Results:**

Weight loss was observed in all infected mice. ATCC 19095 SEC^+^, N315 ST5 TSST-1^+^ and S-70 TSST-1^+^ were arthritogenic, being the highest scores observed in ATCC 19095 SEC^+^ infected mice. Intermediate and lower clinical scores were observed in N315 ST5 TSST-1^+^ and S-70 TSST-1^+^ infected mice, respectively. The ATCC 13565 SEA^+^ strain caused death of 85% of the animals after 48 h. Arthritis triggered by the ATCC 19095 SEC^+^ strain was characterized by accentuated synovial hyperplasia, inflammation, pannus formation, cartilage destruction and bone erosion. Similar joint alterations were found in N315 ST5 TSST-1^+^ infected mice, however they were strikingly more discrete. Only minor synovial proliferation and inflammation were triggered by the S-70 TSST-1^+^ strain. The lowest levels of TNF-α, IL-6 and IL-17 production in response to *S. aureus* stimulation were found in cultures from mice infected with the less arthritogenic strains (S-70 TSST-1^+^ and ATCC 51650 TSST-1^+^). The highest production of IL-17 was detected in mice infected with the most arthritogenic strains (ATCC 19095 SEC^+^ and N315 ST5 TSST-1^+^).

**Conclusions:**

Together these results demonstrated that *S. aureus* strains, isolated from biological samples, were able to induce a typical septic arthritis in mice. These results also suggest that the variable arthritogenicity of these strains was, at least in part, related to their differential ability to induce IL-17 production.

## Background

*Staphylococcus aureus* is a major cause of bacteremia which frequently leads to infective endocarditis, metastatic abscess formation, toxic shock syndrome, gastroenteritis, pneumonia, osteomyelitis and septic arthritis (SA) [1]. The development of these secondary infections is due to bacterial dissemination from the blood to surrounding tissues and is associated with significantly increased morbidity and mortality [1]. Even though all these secondary infections are severe, SA deserves special attention because it is a rapidly progressive and highly erosive disease of the joints that needs an immediate therapeutical intervention [2,3]. The most important risk factor for SA is pre-existing joint disease, especially rheumatoid arthritis (RA) and prosthetic joint surgery [2]. The mortality rate in patients with SA is elevated; around 5-20% of adults with this disease may die as a consequence of their systemic infection [3]. However, in RA patients that have *S. aureus* infections in more than one joint, the mortality risk increases to 50% due to the combination of delayed diagnosis, therapeutic immunosuppression, older age and also the polyarticular involvement [2,3].

One of the hallmarks of SA is the massive inflammation that anticipates bone destruction. The infection by *S. aureus* is accompanied by a rapid recruitment of polymorphonuclear granulocytes and activated macrophages that are then followed by T cells [4]. Although monocytes and macrophages are important to clear bacteria, they also play a pivotal role in the destructive inflammation within the joint [5]. The involvement of pro-inflammatory cytokines in the pathogenesis of *S. aureus* infection has been reported. This bacteria can induce cytokines such as TNF-α, IFN-γ, IL-1, IL-2, and IL-6 [6,7]. Cytokines released from macrophages as TNF-α, IL-1β and IL-6 have been classically pointed as the major players of the severe inflammation that precedes cartilage and bone destruction in SA [2]. The role of IL-17 in SA is not well established. However, a possible deleterious role is highly supported by many reports in the areas of rheumatoid arthritis and osteoarthritis [8]. IL-17A appears to play a key role in host defense against local *S. aureus* infections by inducing the production of neutrophil-mobilizing chemokines, colony-stimulating factors, and cytokines [9].

*S. aureus* strains can produce a number of different components that may contribute to virulence and arthritogenicity, including surface-associated adhesins, capsular polysaccharides, clumping factor A, exoenzymes, and exotoxins [10-12]. Some of the toxins produced by *S. aureus* are called superantigens (SAgs) because they are endowed with the ability to activate various T cell clones, independently of their specificity. These SAgs mediate T cell activation in a very distinctive way from conventional antigens. These molecules are able to simultaneously bind to class II molecules, on antigen presenting cells, and to a large T cell population comprising all clones that share certain variable regions in the TCR Vβ chain [13]. They cause fever, hypotension and other acute toxic-shock-like symptoms by inducing the release of pro-inflammatory cytokines, such as IFN-γ, TNF-α, IL-1 and IL-12 [14,15].

Several studies indicate that experimental staphylococcal arthritis in mice is the best model to study SA because of the striking resemblances between the murine and human immune systems [16,17]. The characteristics of the murine model closely mirror changes seen in human SA, especially with regard to the high frequency and severity of periarticular bone erosivity [4]. LS-1 strain is able to produce TSST-1 and is the most employed *S. aureus* strain to trigger experimental SA [5,16,17]. The main objective of this work was to compare the arthritogenic potential of various SAg-producing staphylococci. Disease incidence, clinical scores, histopathological alterations and cytokine production were the criteria used to characterize arthritis severity.

## Methods

### Experimental design

Mice were infected with different *S. aureus* strains and were daily evaluated by a clinical follow-up that included weight determination, disease incidence and individual clinical scores. Fourteen days after infection they were euthanized and submitted to histopathological and immunological analysis. Cellular immunity was checked considering cytokine production by spleen cells stimulated with *S. aureus* and Concanavalin A (ConA). Non-infected animals were included as a control group. Each group contained 5-9 animals.

### Animals

Male C57BL/6 mice (8-10 weeks old) were purchased from PUSP-RP (USP, São Paulo, SP, Brazil). The animals were fed with sterilized food and water *ad libitum* and were manipulated in accordance with the ethical guidelines adopted by the Brazilian College of Animal Experimentation. All experimental protocols were approved by the local ethics committee for animal experimentation (CEEA), Medical School, Univ. Estadual Paulista (protocol number 291).

### *S. aureus* strains and culture conditions

The following SAg producer strains were used: ATCC 19095 SEC^+^, N315 ST5 TSST-1^+^, S-70 TSST-1^+^, ATCC 51650 TSST-1^+^, and ATCC 13565 SEA^+^. Information related to original isolation and investigations done with these strains is depicted in Table 1. Before each experiment, bacteria were cultured in blood agar plates (Merck) for 24 h at 37°C in order to confirm their purity and to determine their morphology and specific color. Isolated colonies were inoculated in brain heart broth (BHI, Merck) and incubated in 37°C for 24 h. Bacteria were collected by centrifugation, washed three times and resuspended in cold sterile saline, as described by França *et al*., (2009) [18]. The bacterial suspensions were prepared according to the McFarland nephelometer n° 0.5. The exact amount of live bacterial cells was determined by further enumeration of the number of colony forming units (CFU) on agar plates.

**Table 1:** ***S. aureus *strains: origin and infection doses**

**Strains**	**Source**	**Inoculum**	**Reference**
ATCC 19095 SEC^+^	Leg abscess of a patient (Albert Merritt Billings Hospital, University of Chicago, 1933)	2.2x10^7^	[19]
N315 ST5 TSST-1^+^	Pharyngeal smear of a japanese patient, 1982	9.4x10^7^	[20]
S-70 TSST-1^+^	Secretion of a newborn patient (Botucatu, Brazil, 2007)	7.9x10^8^	[21]
ATCC 51650 TSST-1^+^	Wound of a patient with nonmenstrual toxic shock syndrome, Vancouver, British Columbia, Canada	7.8x10^11^	[22]
ATCC 13565 SEA^+^	Food poisoning (Vancouver, Wash, 1940)	1.3x10^7^	[23]

### Arthritis induction

Disease was induced according to the methodology described by Bremell *et al.* (1991), slightly modified [17]. The infection was performed through the retro-orbital route instead of the caudal vein as originally described. Each animal was infected with 0.2 mL of a *S. aureus* suspension made in physiological saline and control mice were injected with 0.2 mL of this diluent. The exact amount of bacteria injected in each group is indicated in Table 1.

### Clinical evaluation

Arthritis was defined as a visible joint erythema and/or swelling of at least one joint. Mice were individually analyzed and joints were inspected every day. The number of arthritic limbs per animal was registered. Arthritis intensity was recorded as described by Abdelnour *et al*. (1993) [24]. Briefly, the arthritic index (clinical score) was carried out by using a system where macroscopic inspection yielded a score of 0–3 points for each limb (1 point = mild swelling and/or erythema; 2 points = moderate swelling and erythema; 3 points = marked swelling and erythema). The clinical score of each group was determined by dividing the total score (sum of the scores of all animals of each group) by the total number of animals in each group.

### Histopathological examination

Joint histopathological examination was done 14 days after infection. After fixation by 10% formaldehyde, the joints were decalcified for 8 weeks in a solution with 18% of ethylenediamine tetraacetic acid. After confirmation that joints were decalcified by a radiographical procedure, they were washed, dehydrated, and embedded in paraffin. Serial sections with 5 μm thickness were cut and stained with haematoxylin and eosin. The sections were semi-quantitatively evaluated in relation to the presence of inflammatory infiltrates, synovial membrane hyperplasia, pannus formation, cartilage destruction and bone erosion.

### Cytokine quantification

Control and infected animals were euthanized 14 days after infection. Spleen cells were collected and adjusted to 5x10^6^cells/mL. Cells were cultured in complete RPMI medium (RPMI supplemented with 5% of fetal calf serum, 20 mM glutamine and 40 IU/mL of gentamicin). Cultures were stimulated with a standardized preparation of *S. aureus* (Pansorbin from Calbiochemical) or ConA (Sigma-Aldrich). Pansorbin is a suspension of heat-killed and formalin-hardened *S. aureus* Cowan I cells and was used at a final dilution of 1:2500 (v:v); ConA was used at a final concentration of 10 μg/mL in the cell culture. Cytokine levels were evaluated 48 h later by enzyme-linked immunosorbent assay (ELISA) in culture supernatants using IFN-γ BD OptEIA Sets (Becton Dickinson) and IL-6, IL-17 and TNF-α Duosets (R&D Systems, Minneapolis, MN, USA). The assays were performed according to the manufacturer’s instruction.

### Statistical analysis

Data were expressed as mean ± SE. Comparisons between infected groups were made by Student’s test or one way ANOVA with Tukey test for parameters with normal distribution. Significance level was p < 0.05. Statistical analysis was accomplished with SigmaStat for Windows v 3.5 (Systat Software Inc).

## Results

### Weight loss

**Figure 1:**
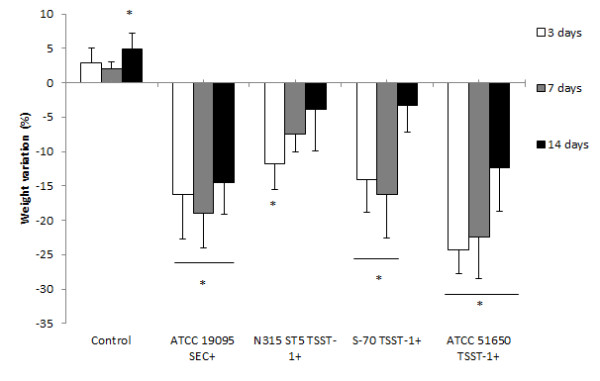
**Weight variation in C57BL/6 mice infected with *S. aureus *strains*.*** Animals were infected with ATCC 19095 SEC^+^, N315 ST5 TSST-1^+^, S-70 TSST-1^+^ or ATCC 51650 TSST-1^+^. Weight variation at 3, 7 and 14 days after infection is illustrated. Data is presented by mean ± SE of 5-9 mice. *represents the difference between initial weight and the weight in each time interval. p < 0.05.

Body weight was daily checked. The percentage of weight variation after 3, 7 and 14 days of infection is illustrated in Figure 1. As can be observed, in spite of some variation, infection with all strains usually determined a significant weight loss.

### Arthritis incidence

**Figure 2:**
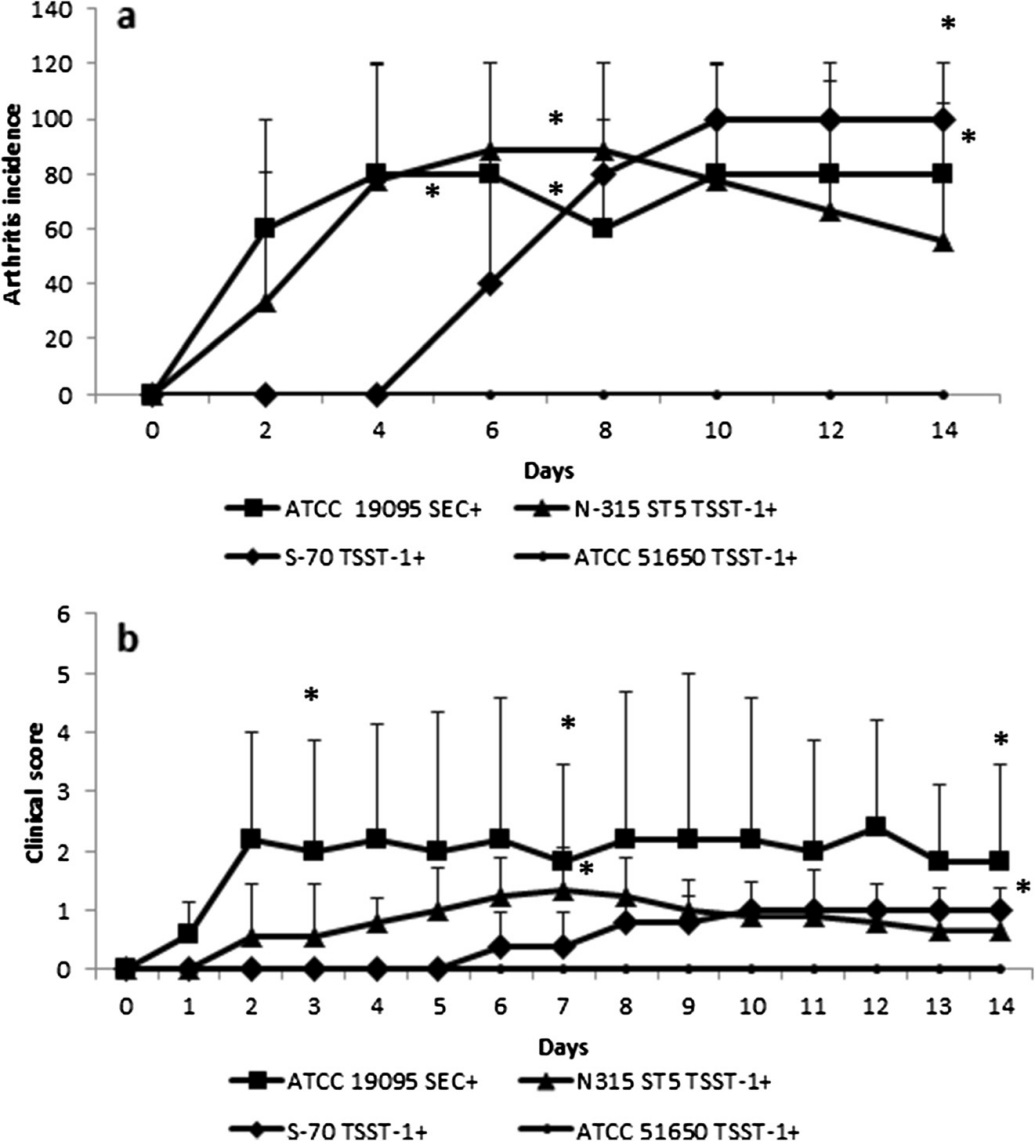
**Arthritis development in C57BL/6 mice infected with S*. aureus *strains*.*** Animals were infected with ATCC 19095 SEC^+^, N315 ST5 TSST-1^+^, S-70 TSST-1^+^ or ATCC 51650 TSST-1^+^. **(a)** Arthritis incidence and **(b)** clinical score were daily evaluated. Data is presented by mean ± SE of 5-9 mice. * represents the difference with the group infected with the non arthritogenic ATCC 51560 TSST-1^+^ strain. p < 0.05.

The kinetics of arthritis development was very similar in the groups infected with ATCC 19095 SEC^+^ and N315 ST5 TSST-1^+^_._ Both groups already presented clinical disease signs (edema and/or erythema) 24 hours after infection. They also reached around 80% of disease incidence. The S-70 TSST-1^+^ infected group presented delayed clinical manifestation that occurred only at the 5th day of infection. A 100% disease incidence was observed in this group at the 10th day of infection. The ATCC 51650 TSST-1^+^ strain did not trigger any sign of arthritis during the planned experimental period. These results are shown in Figure 2a. The ATCC 13565 SEA^+^ strain caused death of 85% of the animals in 48 h (not shown).

### Clinical scores

Clinical scores are shown in Figure 2b. The highest clinical scores were observed in the group infected with the ATCC 19095 SEC^+^ strain. In spite of some variation, these high scores were maintained until the end of the experimental period. Even though hind paws and forepaws were affected, the hind paws presented higher scores (not shown). Lower clinical scores were observed in N315 ST5 TSST-1^+^ and S-70 TSST-1^+^ infected mice. The ATCC 51650 TSST-1^+^ did not provoke any clinical sign of arthritis. The ATCC 13565 SEA^+^ strain was lethal to 85% of the animals, but the remaining 15% did not develop any clinical sign of arthritis.

### Histopathological analysis

**Figure 3:**
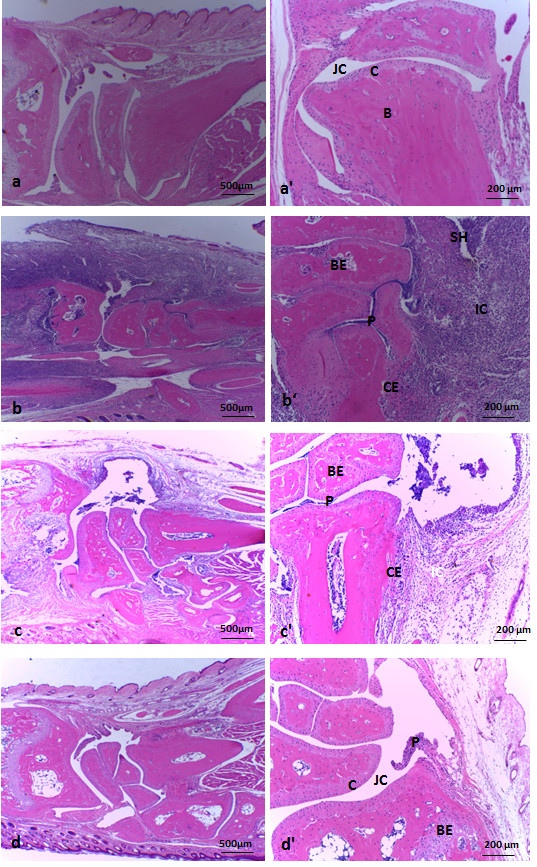
**Histopathological analysis in forepaw joints from C57L/6 mice infected with *S. aureus *strains*. *(a**, **a**’**)** non-infected control. **(b**, **b**’**)** infected with ATCC 19095 SEC^+^. **(c**, **c**’**)** infected with N315 ST5 TSST-1^+^. **(d**, **d**’**)** infected with the S-70 TSST-1^+^. **(a)**, **(b)**, **(c)**, **(d)** and **(a**’**)**, **(b**’**)**, **(c**’**)**, **(d**’**)** represent histopathological micrographics with 4x and 10x magnification, respectively. JC, joint cavity; C, cartilage; B, bone; SH, synovial hyperplasia; BE, bone erosion; CE cartilage erosion; P, pannus formation and IC, inflammatory cells. Panel is representative of 5-9 animals/group.

**Figure 4:**
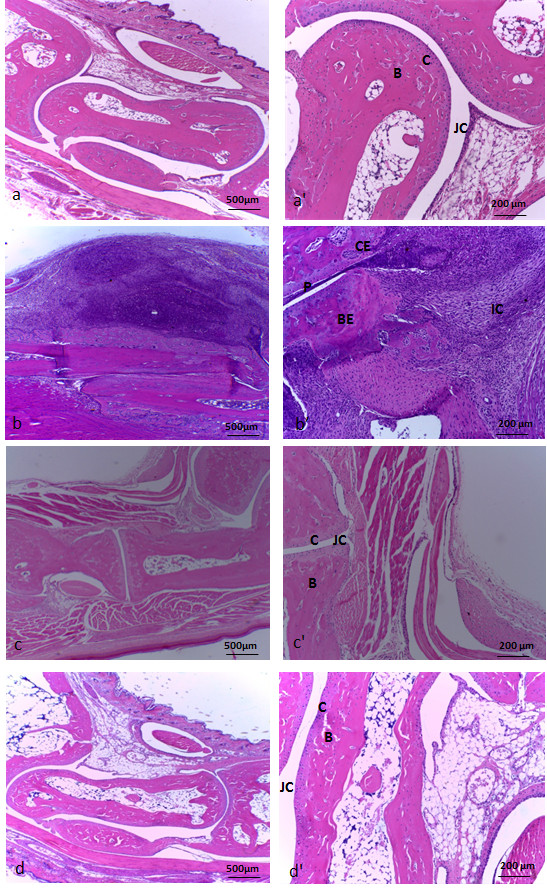
**Histopathological analysis in hind paw joints from C57L/6 mice infected with *S. aureus *strains*. *(a**, **a**’**)** non-infected control. **(b**, **b**’**)** infected with ATCC 19095 SEC^+^. **(c**, **c**’**)** infected with N315 ST5 TSST-1^+^. **(d**, **d**’**)** infected with the S-70 TSST-1^+^. **(a)**, **(b)**, **(c)**, **(d)** and **(a**’**)**, **(b**’**)**, **(c** ’**)**, **(d**’**)** represent histopathological micrographics with 4x and 10x magnification, respectively. JC, joint cavity; C, cartilage; B, bone; BE, bone erosion; CE cartilage erosion; P, pannus formation and IC, inflammatory cells. Panel is representative of 5-9 animals/group.

The normal histological pattern of mice joints is shown in Figures 3a and 3a’. A very thin synovial layer, a marked joint cavity, an intact cartilage and a preserved bone structure can be observed. After 14 days of infection the histological aspect of swollen joints from forepaws was very distinct in the three experimentally infected groups. Mice infected with the ATCC 19095 SEC^+^ strain presented a severe arthritis characterized by a marked proliferation of synovial tissue and a huge inflammatory process. The inflammatory infiltrate was mainly localized inside the synovial cavity but it also penetrated into the cartilage and the bone. A striking pannus formation occupied almost the entire joint cavity. Cartilage and bone erosion were also observed in many areas (Figures 3b and 3b’). Synovial hyperplasia, inflammation, pannus formation and cartilage and bone erosion were also detected in mice infected with the N315 ST5 TSST-1^+^ strain but in lesser extension (Figures 3c and 3c’). As shown in Figures 3d and 3d’, only minor degrees of synovial proliferation and inflammation were detected in the joints of mice infected with the S-70 TSST-1^+^ strain. Arthritis in the hind paws of ATCC 19095 SEC^+^ infected animals was even more severe than in the forepaws. In this case, the synovial space was entirely occupied by inflammatory infiltrates and bone tissue was severely eroded (Figures 4b and 4b’). Various cell types as polymorphonuclear cells, mononuclear cells and fibroblast-like cells were observed. Granuloma-like structures were also visualized in these joints. Histopathological changes were not detected in the hind paws of animals infected with N315 ST5 TSST-1^+^ or S-70 TSST-1^+^*S. aureus* strains as can be observed in Figures 4c and 4d*.*

### Cytokine production

**Figure 5:**
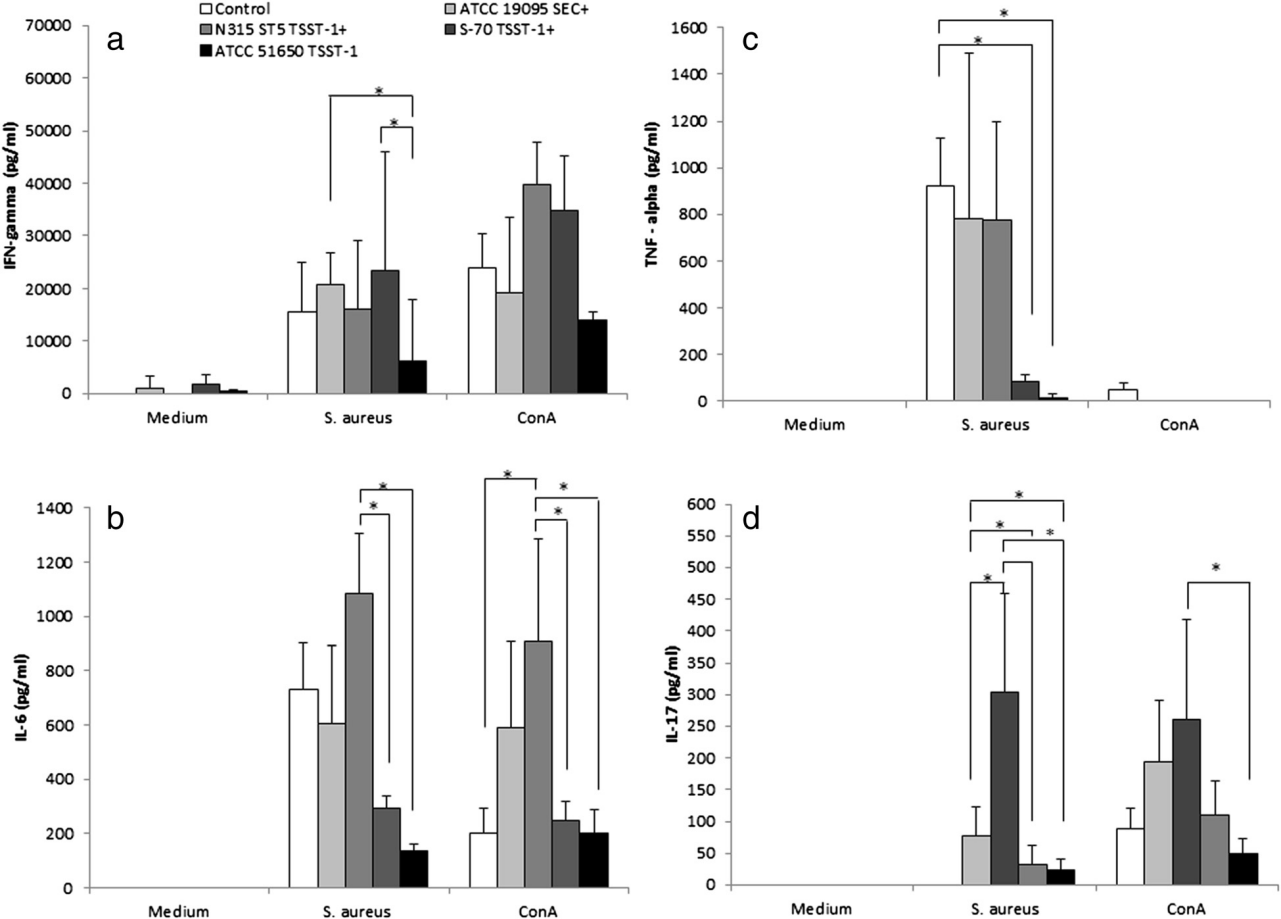
**Production of cytokines by spleen cell cultures stimulated with *S. aureus *and ConA.** C57BL/6 mice were infected with ATCC 19095 SEC+, ATCC N315 ST5 TSST-1+, ATCC S-70 TSST-1+, and ATCC 51650 TSST-1+. IFN-γ **(a)**, IL-6 **(b)**, TNF-α **(c)** and IL-17 **(d)** were quantified in culture supernatants by ELISA after 48 h of incubation. Data is presented by mean ± SE of 5-9 mice. *represents statistical difference between the two indicated groups. p < 0.05.

The cellular immune response was evaluated by the production of cytokines by spleen cells stimulated with fixed *S. aureus* Cowan strain I or ConA. High levels of IFN-γ were produced by cells stimulated with *S. aureus*. Even though these levels were generally similar to the ones produced by cells from control (non-infected) mice, the ATCC 19095 SEC^+^ and S-70 TSST-1^+^ infected groups produced significantly higher amounts than the ATCC 51650 TSST-1^+^ group (Figure 5a). Stimulation with ConA triggered elevated IFN-γ production by all experimental groups including, as expected, the control group (Figure 5a). All groups produced TNF-α when the cells were stimulated with *S. aureus*, being the levels found in the control, ATCC 19095 SEC^+^ and N315 ST5 TSST-1^+^ similarly elevated. The levels of this cytokine were, however, significantly lower in the groups S-70 TSST-1^+^ and ATCC 51650 TSST-1^+^ (Figure 5c). Production of IL-6 induced by *S. aureus* was significantly lower in the group infected with *S. aureus* S-70 TSST-1^+^ (Figure 5b). A very similar profile was observed when the cultures were stimulated with ConA, i.e., significantly lower levels of IL-6 were produced by mice infected with S-70 TSST-1^+^ and ATCC 51650 TSST-1^+^ strains in comparison to N315 ST5 TSST-1^+^ (Figure 5b). The profile of IL-17 production was similar in the cultures stimulated with *S. aureus* and ConA, being its levels higher in cultures from N315 ST5 TSST-1^+^ and ATCC 19095 SEC^+^ groups as can be observed in Figure 5d. The most striking differences were, however, observed in the cultures stimulated with *S. aureus*. In this case, IL-17 levels were significantly higher in the groups N315 ST5 TSST-1^+^ and ATCC 19095 SEC^+^ in comparison to all other groups (Figure 5d).

## Discussion

Septic arthritis is an infectious disease that affects the joints. Due to its fast evolution, even with prompt therapy, it can cause irreversible joint damage and even death [2]. *S. aureus* is the most common causative agent of this disease and it has been believed that SAg-production plays a pivotal role in arthritogenicity [2,15]. The main goal of this work was to compare the arthritogenic potential of SAg-producing *S. aureus* strains considering disease incidence, clinical score and histopathological alterations. Cytokine production was also evaluated to get some insight into possible differences among the various strains. An initial screening process was performed to evaluate susceptibility of C57BL/6 and BALB/c mice to develop septic arthritis after *S. aureus* infection. Gender effect was also checked. This preliminary evaluation, that was done only with the ATCC 19095 SEC^+^ strain, indicated that male and female, from both mice strains, lost a significant percentage of weight in the first days of infection. We believe that all animals were similarly infected because weight loss is accepted as a parameter to indicate effective infection [25]. However, only C57BL/6 male mice developed arthritis. This resistance of BALB/c mice to develop experimental arthritis was already described by Bremmel *et al*. (1991) [17]. Our results were, however, distinct from the more recently published data of Henningsson et al. (2010) [26]. These authors described a high incidence of arthritis in both male and female C57BL/6 mice infected with the LS-1 TSST-1^+^*S. aureus* strain. This variable susceptibility could be related, at least partially, to *S. aureus* strain particularities. C57BL/6 male mice were, therefore, chosen to compare the arthritogenic potential of the different *S. aureus* strains. These animals were then infected by the retro-orbital plexus and body weight and clinical scores were daily checked until the 14th day when animals were euthanized for histopathological and immunological evaluations. The fact that inoculation of all 5 strains triggered significant weight loss, suggests successful experimental infections as has been proposed by other authors [25].

Arthritis incidence and clinical scores varied among *S. aureus* strains. ATCC 19095 SEC^+^ and N315 ST5 TSST-1^+^ were the most arthritogenic ones. They triggered earlier symptoms and the highest levels of incidence. The ATCC 19095 SEC^+^ strain was associated with the highest clinical scores. The S-70 TSST-1^+^ infected group presented a delayed clinical manifestation and lower clinical scores whereas the ATCC 51650 TSST-1^+^ strain did not cause any sign of arthritis during 14 days. The ATCC 13565 SEA^+^ provoked death of 85% of the animals after 48 h (not shown). If these differential outcomes in mice can be translated to human SA is a subject that needs further investigation. Many bacterial components are thought to contribute to arthritogenicity [10-12]. The delayed appearance of arthritis in S-70 TSST-1^+^ infected mice could mean, for example, that this strain is endowed with a smaller number of arthritogenic factors than the other ones. In this scenario, we could think that this strain is being better controlled by the innate immunity. Alternatively, some strains, as seems to be the case of S-70 TSST-1^+^, could be less inflammatory. The level of pro-inflammatory cytokines showed by our results indicates that this less arthritogenic strain induced lower levels of TNF-α, IL-6 and IL-17 than the two more arthritogenic ones.

No direct relationship was found between arthritogenicity and bacterial inoculum. By comparing bacterial inoculum with the degree of arthritis severity, we can conclude that the differential arthritis severity was not associated with distinct bacterial concentrations. We cannot rule out, however, the possibility that these strains present distinct potential to colonize the joints. Unexpectedly, even though the bacterial suspensions from the 5 strains were prepared by the same methodology, they really contained distinct number of viable bacteria. This subject was not further evaluated but it suggests that these preparations could have distinct proportions of dead and alive bacteria. Concerning this differential arthritogenicity among *S. aureus* strains we would like to highlight two aspects. This is the first demonstration that these strains, that were originally isolated from biological samples, can cause septic arthritis in mice. In addition, these results indicate that superantigenicity was not enough to elicit arthritis. The ATCC 51650 TSST-1^+^ strain, for example, was not able to induce septic arthritis, even though it was used in the adequate range of bacterial concentration [16]. This finding is in accordance with the postulate that induction of arthritis by *S. aureus* infection is likely elicited by the concerted action of multiple events as activation of T lymphocytes by SAgs, exposure to peptidoglycans/capsular polysaccharides, presence of surface-associated adhesins, clumping factor A and also free bacterial DNA [2,10-12]. Even though not clearly demonstrated in the literature, the ability of *S. aureus* to form biofilms with joint components is being suggested as a possible arthritogenic element [27,28]. In this sense, it would be very enlightening to compare the ability of these *S. aureus* strains to form biofilms.

The histopathological analysis of the joints revealed the presence of synovial proliferation, pannus formation and inflammatory infiltrates in the joint cavity. These findings are similar to the most consensual features of SA caused by *S. aureus*[29]. Cartilage and bone erosion were also present, mainly in joints from animals infected with the ATCC 19095 SEC^+^ strain. This evolution from the inflammatory process to cartilage and bone erosion is very relevant because it mimicries the human situation during SA by *S. aureus*. In this case, 25-50% of the patients progress to bone destruction and irreversible loss of joint function [30-32].

TNF-α, IL-6 and IL-17 are being described as some of the most relevant mediators in SA immunopathogenesis [26,33]. To investigate the possible contribution of cytokines to these histopathological alterations, the production of TNF-α, IFN-γ, IL-6 and IL-17 was simultaneously quantified in spleen cultures stimulated with *S. aureus* or ConA. Even though all of them can be released during the initial innate immunity, IFN-γ and IL-17 can also be produced during specific immunity by effector Th1 and Th17 cells, respectively. Interestingly, the lowest levels of TNF-α, IL-6 and IL-17 were found in cultures from mice that presented the lowest clinical scores. In addition, differently from IFN-γ, TNF-α and IL-6, IL-17 was not produced by spleen cells from normal mice. The highest production of IL-17 was observed in infections caused by the more arthritogenic strains, i.e., ATCC 19095 SEC^+^ and N315 ST5 TSST-1^+^. The role of IL-17 as a mediator of joint destruction is being elucidated in experimental RA. The intra-articular injection of IL-17 into the knee results in joint inflammation and local damage [32]. This effect has been attributed, at least partially, to IL-17 induction of matrix metalloproteinases and also to its ability to promote osteoclastogenesis [34]. IL-17 also induces production of IL-6 and IL-8 by RA synovial fibroblasts via NF-kB and PI3-kinase/Akt-dependent pathways [35]. Furthermore, this cytokine induces production of chemokines and other pro-inflammatory cytokines such as TNF-α, IL-1β, CXCL1 and CXCL5 [36,37]. These cytokines affect bone remodeling by stimulating proliferation and differentiation of osteoclast progenitors into mature osteoclasts [38,39]. Contrasting with this well-established role of IL-17 in RA, the role of IL-17 in *S. aureus*-induced arthritis is not well understood. To our knowledge, this higher production of IL-17 in mice infected with the most arthritogenic strains is being described for the first time. Although this cytokine has been associated with protection in animals immunized with clumping factor A and also with local host defense during *S. aureus*-induced arthritis, its arthritogenic contribution in SA is still not disclosed. In this sense, these results support the possibility that higher IL-17 inducer *S. aureus* strains are endowed with stronger arthritogenic abilities [26,40].

## Conclusions

Altogether, these results demonstrated that *S. aureus* strains, isolated from biological samples, were able to induce typical septic arthritis in mice. These results also suggest that the variable arthritogenicity of these strains was, at least in part, related to their differential ability to induce IL-17 production.

## Abbreviations

ATCC: American type culture collection; BHI: Brain heart infusion; CFU: Colony forming units; ConA: Concanavalin A; NF-kB: Nuclear factor kappa B; RA: Rheumatoid arthritis; SA: Septic arthritis; SAg: Superantigen; SEC: Staphylococcal enterotoxin C; SEA: Staphylococcal enterotoxin A; TSST: Toxic shock syndrome toxin; CEEA: Ethcis committee for animal experimentation; LS-1: *Paracoccus seriniphilus* sp. nov.; ELISA: Enzyme linked immunosorbent assay; SE: Standard error; BD: Becton Dickinson and Company; R&D: Research and development.

## Competing interests

The authors declare that they have no competing interests.

## Authors’ contributions

This study was conceived by PMCM and AS. All authors contributed to carry out the experiments, read and approved the final manuscript.

## Pre-publication history

The pre-publication history for this paper can be accessed here:

http://www.biomedcentral.com/1471-2334/13/400/prepub
